# Sex-based associations between neighborhood disadvantage and brain–gut alterations in individuals with irritable bowel syndrome

**DOI:** 10.1186/s13293-025-00739-y

**Published:** 2025-08-11

**Authors:** Lisa A. Kilpatrick, Lin Chang, Jennifer S. Labus, Andrea S. Shin, Michelle Choy, Tien S. Dong, Bruce Naliboff, Emeran A. Mayer, Arpana Church

**Affiliations:** 1 Vatche and Tamar Manoukian Division of Digestive Diseases, University of California Los Angeles, Los Angeles, CA USA; 2 Goodman-Luskin Microbiome Center, University of California Los Angeles, Los Angeles, CA USA; 3 G. Oppenheimer Center for Neurobiology of Stress and Resilience, University of California Los Angeles, Los Angeles, CA USA; 4 Department of Medicine, David Geffen School of Medicine, University of California Los Angeles, Los Angeles, CA USA; 5 David Geffen School of Medicine, University of California Los Angeles, Los Angeles, CA USA; 6 Brain Research Institute, Gonda (Goldschmied) Neuroscience and Genetics Research Center, University of California Los Angeles, Los Angeles, CA USA

**Keywords:** Irritable bowel syndrome, Sex-differences, Brain–gut–microbiome interactions, *Prevotella*, Area deprivation index

## Abstract

**Background:**

Irritable bowel syndrome (IBS) is a stress-sensitive disorder that exhibits sex differences in brain–gut–microbiome interactions. Neighborhood disadvantage is a chronic stressor that may influence brain–gut–microbiome health in patients with IBS, potentially contributing to clinical profiles in a sex-specific manner. This study evaluated sex-based associations between neighborhood disadvantage and clinical characteristics, cortical morphology, and *Prevotella* relative abundance (a sex-specific microbial marker in IBS) in individuals with IBS compared to healthy controls (HCs).

**Methods:**

Brain magnetic resonance imaging scans were obtained in 182 individuals with IBS (age, 31.0 ± 0.8 years; 128 females) and 161 HCs (age, 32.7 ± 1.0 years; 94 females). Fecal microbiome data was available in 113 IBS participants (80 females) and 127 HCs (74 females). Current neighborhood disadvantage was assessed as the Area Deprivation Index (ADI), with ADI⩾5 defined as high ADI. Group differences in the associations of high ADI with symptoms, *Prevotella*, and cortical morphology were evaluated using partial least squares.

**Results:**

Diagnosis Differences: High ADI was associated with greater lateral intraparietal surface area in IBS vs HCs. Sex Differences: There were greater negative associations between high ADI and surface area in frontal operculum and thickness in frontopolar and primary somatosensory regions in females vs males. Diagnosis*Sex Differences: There were greater negative associations between high ADI and surface area in superior parietal and sensorimotor regions in IBS females vs males, and greater negative associations between high ADI and surface area and thickness in dorsolateral prefrontal and parietal regions, respectively, in IBS males vs females. High ADI was associated with greater symptom severity in IBS males, greater perceived stress in both IBS and HC females, and *Prevotella* relative abundance in IBS females (all p’s < 0.01).

**Conclusions:**

Neighborhood disadvantage is associated with greater symptom severity in IBS males and both higher perceived stress (exacerbates symptoms) and *Prevotella* abundance (protective) in IBS females. It generally has a greater negative impact on emotion/pain-related cortical morphology in females vs males. However, there are more prominent somatosensory reductions in IBS females, and prefrontal reductions in IBS males. These findings highlight the interplay between social and biological factors in IBS and underscore the need for targeted, sex-specific interventions.

**Graphical abstract:**

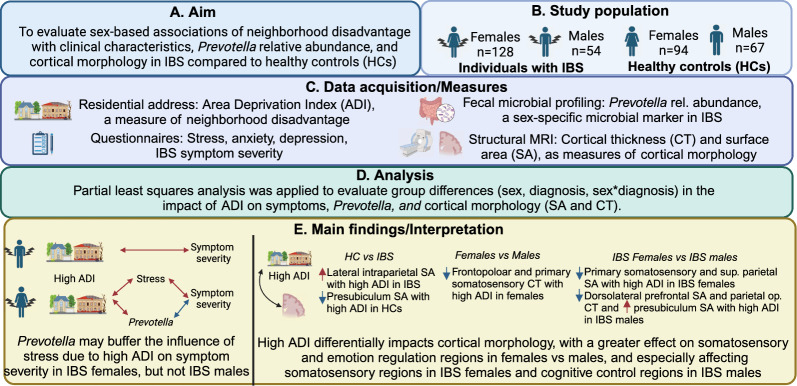

## Abstract (plain language summary)

Irritable bowel syndrome (IBS) is a stress-sensitive disorder that is more common in females than in males and has been linked to changes in the brain–gut microbiome system. Living in a disadvantaged neighborhood is a type of chronic stressor that could contribute to changes in the brain–gut microbiome system and symptoms in IBS. Here, we investigated whether the area deprivation index, a measure of neighborhood disadvantage (based on neighborhood rates of poverty, unemployment, etc.), is associated with differences in symptoms, stress, brain structure, and the level of *Prevotella* in the gut in males and females with IBS and healthy individuals. Based on our results, neighborhood disadvantage negatively affects the structure of brain regions involved in emotion and pain processing to a greater extent in females than in males; this may contribute to a greater risk of IBS in females, but additional research is needed. Neighborhood disadvantage is associated with additional changes in somatosensory brain regions (involved in pain processing) in females with IBS and cognitive control brain regions (involved in pain modulation) in males with IBS. We also found that high neighborhood disadvantage is associated with worse symptom severity in males with IBS, and with greater stress and *Prevotella* in females with IBS. As stress is positively associated with symptoms while *Prevotella* is negatively associated with symptoms in females with IBS, both risk (stress) and protective factors (*Prevotella*) may be present in disadvantaged neighborhoods. Understanding how these factors affect individuals with IBS can aid in improving treatment effectiveness.

## Highlights

- Neighborhood disadvantage is associated with increased symptom severity in males with IBS.
- Neighborhood disadvantage is associated with increased stress and *Prevotella* relative abundance in females with IBS, which have opposing relationships with symptom severity, suggesting that sex-specific risk (stress) and protective factors (*Prevotella*) exist in disadvantaged neighborhoods.
- Neighborhood disadvantage negatively affects the morphology of brain regions involved in emotion and pain processing to a greater extent in females than in males.
- Neighborhood disadvantage shows additional sex-specific associations in IBS, with more prominent somatosensory alterations in females with IBS and cognitive control alterations in males with IBS.

## Introduction

Irritable bowel syndrome (IBS) is a female-predominant disorder of gut–brain interactions, characterized by abdominal pain and alterations in stool form and/or frequency [1]. Sex differences exist in the clinical characteristics; females with IBS have more severe symptoms and comorbid anxiety/depression than males with IBS [2–4]. Although the structure and function of brain regions involved in emotion and pain processing are affected in both males and females with IBS, sex differences have been noted [5]. In particular, females with IBS compared to males with IBS show more prominent somatosensory alterations, which are associated with increased symptom severity [5, 6].

Chronic, repeated stress can increase the risk of developing IBS, as well as increase the severity of disease-related symptoms among those with IBS [7]. Chronic stress can impact interactions between components of the brain–gut–microbiome axis relevant to IBS pathophysiology [8, 9]. For instance, chronic stress is associated with changes in brain structure and function resulting in altered emotion and pain processing and increased stress responsiveness, which can in turn affect the immune system and gut microbiome composition [8, 10, 11]. Sex differences in the impact of chronic stress on components of the brain–gut–microbiome axis have also been frequently reported [10, 12, 13].

Conditions associated with neighborhood disadvantage, including high poverty, unemployment, and crime rates; exposure to pollution and toxins; physical and social disorder; and lack of resources such as healthcare, greenspace, and healthy foods, can be conceptualized as stressors constraining an individual’s ability to adapt and thrive [14]. Consistent with this, living in a disadvantaged neighborhood is associated with psychological distress and poor self-reported health [14–16], as well as with worse outcomes in patients with chronic conditions, including chronic pain [17, 18]. Neighborhood disadvantage in adulthood is associated with increased pain sensitivity [18] and altered brain morphology overlapping IBS-implicated regions, including regions involved in stress responsiveness and pain processing [19, 20]. Further, neighborhood disadvantage can shape the gut microbiome composition [21, 22]. In particular, neighborhood disadvantage is associated with increased *Prevotella* abundance [21, 22]. Several studies have reported differences in *Prevotella* abundance between individuals with IBS and healthy controls (HCs), with most indicating lower levels in IBS [23, 24]; however, a study conducted in China reported increased *Prevotella* in IBS [25]. Differences in the neighborhood context may contribute to these differences in results. Further, in our previous study, we identified *Prevotella* relative abundance as a sex-specific microbial marker in IBS, with lower *Prevotella* relative abundance associated with greater symptom severity in females, but not males, with IBS [26]. Thus, increased *Prevotella* abundance with high neighborhood disadvantage may be protective against other factors associated with high neighborhood disadvantage that contribute to symptom severity (such as stress) in females with IBS. Overall, these findings suggest that neighborhood disadvantage may influence brain–gut–microbiome health in patients with IBS in a complex, sex-specific manner. Understanding how broad, modifiable socioeconomic contexts such as neighborhood disadvantage impact brain–gut–microbiome mechanisms and clinical profiles in IBS is crucial for improving the quality of life and addressing barriers to effective treatment.

**Fig. 1:**
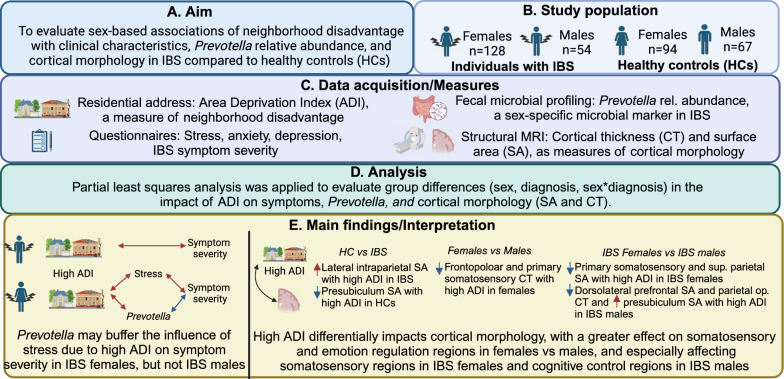
Graphical abstract. This study investigated associations between the area deprivation index, a measure of neighborhood disadvantage, and symptoms, stress, brain morphology, and *Prevotella* relative abundance in males and females with IBS and healthy individuals. We found that neighborhood disadvantage is associated with increased symptom severity in males with IBS, and both perceived stress and *Prevotella* relative abundance in females with IBS, which have positive and negative relationships with symptom severity, respectively. Additionally, neighborhood disadvantage mainly has a negative impact on cortical morphology, with greater reductions in emotion/pain-related regions in females vs males, somatosensory regions in IBS females vs IBS males, and cognitive control regions in IBS males vs IBS females

Therefore, the present study evaluated sex-based associations of neighborhood disadvantage with cortical morphology, *Prevotella* relative abundance, and clinical characteristics in individuals with IBS compared to HCs (Fig. 1). We hypothesized that neighborhood disadvantage would be associated with greater perceived stress; greater anxiety and depression symptoms in IBS (especially in females with IBS); greater IBS symptom severity in participants with IBS; greater alterations in the cortical morphology of regions implicated in IBS; and greater *Prevotella* relative abundance in females, but not males, with IBS.

## Methods

### Participants

Participants comprised 182 males and females with IBS (128 females; 54 males) and 161 HCs (94 females; 67 males) who underwent brain magnetic resonance imaging (MRI) at the University of California, Los Angeles (UCLA) from 2013 to 2017 and from 2020 to 2024. Fecal microbiome data were available in a subset of these participants, including 113 participants with IBS (80 females; 33 males)) and 127 HCs (74 females; 53 males). All participants provided a residential address. Participants were recruited by flyers posted on UCLA campus and doctor offices in the Los Angeles area, as well as by mass emails to the UCLA community and listings on social media.

All participants with IBS were evaluated by a clinician with expertise in IBS to determine fulfillment of Rome III (for participants imaged from 2013 to 2017) or Rome IV diagnostic criteria (for participants imaged from 2020 to 2024) [27, 28]. All bowel habit types were included. HCs comprised healthy individuals without a history of IBS. The following exclusion criteria were applied to both HCs and participants with IBS: major neurological condition; gastric, abdominal, or colon surgery; inflammatory bowel disease; ulcer disease; vascular disease; current or past psychiatric illness; alcohol or drug misuse (occasional recreational use was allowed, but participants were asked to refrain from use during the study); use of medications that interfere with the central nervous system (e.g. antidepressants, neuromodulators); pregnant or breastfeeding; excessive physical exercise (> 8 h/week); and MRI contraindications.

All participants provided written informed consent. This study was approved by the Institutional Review Board at the University of California, Los Angeles’s Office of Protection for Research Subjects (Nos. 20-000540, 20-000515, 20-0540, 20-0515).

### Questionnaire-based assessments

Basic demographic information data was obtained from all participants. In addition, all participants completed the Hospital Anxiety and Depression (HAD) scale and Perceived Stress Scale (PSS). The HAD assesses current anxiety and depression symptoms (within the prior month), and has a total score ranging 0–21, with higher scores reflecting greater anxiety/depression [29]. The Perceived Stress Scale (PSS) assesses feelings of stress within the prior month, and has a total score ranging 0–40, with higher scores reflecting greater stress [30]. Participants with IBS also completed the IBS Severity Scoring System (IBS-SSS), as a measure of symptom severity [31]. The IBS-SSS has a total score of 0–500, with scores of 175–300 reflecting moderate symptom severity and scores > 300 reflecting severe symptom severity [31].

In addition, participants completed a validated dietary questionnaire [32], in which participants were asked to indicate the dietary pattern that best reflected their diet from a descriptive list of major dietary patterns (e.g., standard American, Mediterranean, vegetarian). As in our previous research, diets were then categorized as either American (standard American or modified American), Mediterranean, vegetarian (vegan or vegetarian) or restrictive (low FODMAP [fermentable oligo-, di-, and monosaccharides and polyols], gluten free, or dairy free) [26].

### Assessment of neighborhood disadvantage

**Fig. 2:**
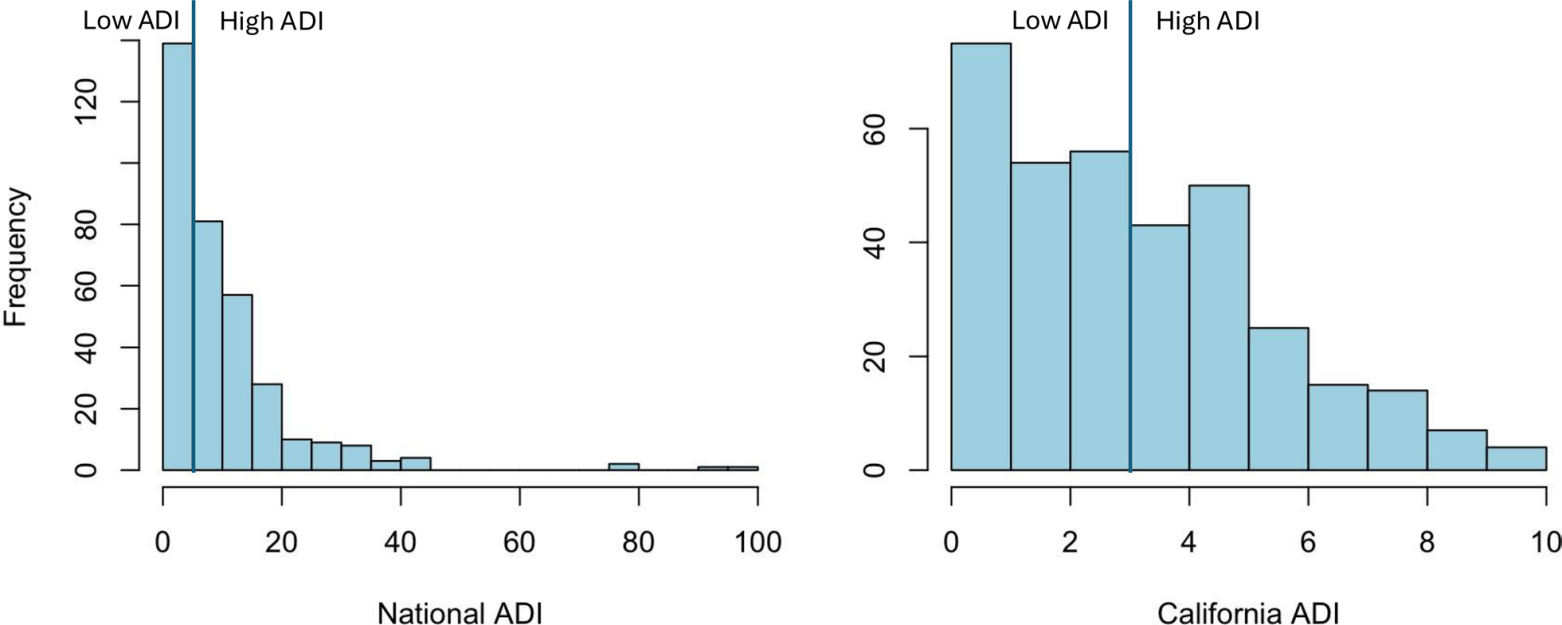
Distribution of National and California ADI in the present sample. The National ranking is provided in percentiles (range, 1–100), while the California state-wide ranking is provided in deciles (range, 1–10). The cutoff for low/high ADI is shown according to both National and California ranking. It is important to note that although the cutoff results in comparisons between participants living in the lowest ~ 30% to the highest ~ 70% of neighborhoods in terms of the California ADI, the low ADI group represents extremely low ADI at the National level. This is due to the relatively low ADI estimates in southern California compared to those in other area of the nation. *ADI* area deprivation index

Neighborhood disadvantage was assessed using the Neighborhood Atlas [33]. The Neighborhood Atlas estimates neighborhood disadvantage as a composite of 17 factors reflecting the average income, educational attainment, employment, and housing quality in a neighborhood [33], based on U.S. Census Bureau and American Community Survey data. The Neighborhood Atlas provides the Area Deprivation Index (ADI) at the national level, with neighborhoods nationally ranked by percentiles, and the state level, neighborhoods ranked within the state by deciles, with higher scores indicating greater neighborhood disadvantage. Recent reports indicate high internal reliability for the ADI, with a Cronbach's alpha of 0.93 [34]. As the participant data were obtained over two time periods (from 2013 to 2017 and from 2020 to 2024), we used archival 2015 ADI data for scans collected from 2013 to 2017 and 2020 ADI for scans collected from 2020 to 2024. As all participants resided in California, we evaluated both state and national ADI. We dichotomized ADI at the national level to compare neighborhoods with low levels of disadvantage (lowest ~ 1/3 in ADI rankings) to neighborhoods with higher levels of disadvantage (highest ~ 2/3 in ADI rankings). Given the distribution of ADI in the study sample, which was skewed towards lower ADI (Fig. 2), high ADI was defined as a national-level ADI of at least 5, which roughly corresponded to a state-level ADI of at least 3. Thus, in the present study, ‘low ADI’ represents neighborhoods with the ~ 30% lowest ADI and ‘high ADI’ represents neighborhoods with the highest ~ 70% ADI of all neighborhoods in California, which generally have low ADI compared to other states.

### Assessment of Prevotella relative abundance

A subset of participants provided stool samples for fecal microbial profiling. Stool samples were collected using a home stool collection kit. HCs were asked to avoid providing samples under acute constipation or diarrhea. All participants were instructed to preserve stool samples in a freezer immediately after collection, until they could be delivered to a study coordinator or collected by a courier service within 24–48 h of collection. Samples were subsequently stored at –80°C until processing, which entailed grinding them into a coarse powder using a mortar and pestle under liquid nitrogen and aliquoting them for further analysis.

Fecal microbial profiling was conducted as previously reported [26]. Briefly, the Qiagen Powersoil DNA Isolation Kit (MO BIO Laboratories) was used for DNA extraction. A sequencing library was then created by amplifying the V4 hypervariable region of the 16S ribosomal RNA gene using 515F and 806R primers similar to previously published protocols [35, 36]. Sequencing (2 × 250) on an Illumina HiSeq 2500 was performed. The DADA2 R package was used to generate tables of denoised amplicon sequence variants, which were subsequently merged [37]. Taxonomic assignments were performed using the SILVA 138.1 database. Differential abundance testing was performed using DESeq2 in R, employing an empirical Bayesian approach to shrink dispersion and fit non-rarified count data to a negative binomial model [38]. From this, the relative abundance of *Prevotella* was calculated.

### Neuroimaging acquisition and preprocessing

As cortical thickness and surface area are influenced by different biological processes, and cortical volume represents a combined metric, we decided to focus on cortical thickness and surface area in the present study [39, 40]. High-resolution T1-weighted scans were obtained on a 3.0 T Siemens Prisma MRI scanner (Siemens, Erlangen, Germany), with the following parameters: echo time, 1.81 ms; repetition time, 2500 ms; slice thickness, 0.8 mm; number of slices, 208; voxel matrix, 320 × 300; and voxel size, 1.0 × 1.0 × 0.8 mm. Imaging data were preprocessed using fMRIprep (version xx) [41]. Briefly, T1-weighted images underwent skull-stripping, followed by segmentation, cortical surface reconstruction, and parcellation according to the HCPMMP 1.0 atlas [42] using FreeSurfer 6.0 [43]. Mean cortical thickness and surface area were then extracted for all 360 cortical regions in the HCPMMP 1.0 atlas.

### Statistical analysis

For demographic data, means along with their standard errors are reported, and comparisons between means were conducted using analysis of variance. Categorical data were analyzed using Pearson’s chi-squared test.

Firstly, non-rotated partial least squares (PLS) analysis was utilized to identify brain morphologic profiles commonly and differentially associated with ADI according to sex and diagnosis. The analysis was performed with adjustment for age, education, and intracranial volume by residualizing the data. PLS was implemented using freely available code (http://www.rotman-baycrest.on.ca/pls) [44]. A priori contrasts were constructed to identify (1) disease-related differences between participants with IBS and HCs in terms of the effect of ADI on morphology; (2) sex-related differences in the effect of ADI on morphology; and (3) effects of ADI on morphology dependent on both sex and diagnosis (i.e. sex*diagnosis interaction). The reliability of group differences in the relationship between ADI and brain morphology (360 cortical regions) was determined by 5000 bootstrap samples. Bootstrap ratios with a magnitude > 2.58 (corresponding to p < 0.01) were considered reliable.

Secondly, symptom-microbial profiles associated with ADI were evaluated in the subset of participants with gut microbiome data, using PLS analysis. As the variables of interest and controlled variables differed between participants with IBS and HCs, separate analyses were performed. Specifically, an analysis of symptom-microbial profiles associated with ADI in male and female participants with IBS included the following variables of interest: IBS-SSS, HAD anxiety, HAD depression, PSS, and *Prevotella* relative abundance, with adjustment for age, education, bowel habit, and diet. A similar analysis of symptom-microbial profiles associated with ADI in male and female HCs included the following variables of interest: HAD anxiety, HAD depression, PSS, and *Prevotella* relative abundance, with adjustment for age, education, and diet. The reliability of the relationship between ADI and symptoms and *Prevotella* relative abundance was determined by 5000 bootstrap samples. Bootstrap ratios with a magnitude > 2.58 (corresponding to p < 0.01) were considered reliable.

A final analysis was performed to relate morphological results to clinical/microbial results in participants with IBS. Relationships between cortical morphology identified as differentially associated with ADI (first analysis) and clinical/microbial parameters identified as associated with ADI (second analysis) were evaluated in the subset of participants with IBS and gut microbiome data using PLS analysis, with adjustment for age, education, bowel habit, intracranial volume, and diet. Bootstrap ratios with a magnitude > 1.96 (corresponding to p < 0.05) were considered reliable.

## Results

### Demographic and clinical characteristics

Demographic and background characteristics of the participants are shown in Table 1. HAD anxiety, HAD depression, and PSS scores were significantly higher in participants with IBS than in HCs (p’s < 0.001) (Table 1). HAD anxiety also showed a significant main effect of sex (p < 0.001) and a significant diagnosis*sex interaction (p = 0.01), with higher scores in females than in males, especially in females with IBS (Table 1). Diet also showed a significant main effect of sex (p = 0.01) and a significant diagnosis*sex interaction (0.01), with American diet more common in males than in females, especially males with IBS (Table 1).

**Table 1:** Participant characteristics

	Females with IBS	Males with IBS	Female HCs	Male HCs	P-values		
					Dx	Sex	Dx*Sex
n	128	54	94	67			
Age (Yrs)	30.3 (0.9)	32.5 (1.3)	33.6 (1.5)	31.9 (1.2)	0.14	0.72	0.15
National ADI (1–100)	11.1 (1.2)	11.6 (1.4)	9.7 (1.3)	9.9 (1.2)	0.22	0.85	0.98
California ADI (1–10)	3.7 (0.2)	3.9 (0.3)	3.4 (0.2)	3.6 (0.3)	0.15	0.58	0.93
HAD-Anx (0–21)	7.4 (0.3)	7.4 (0.5)	4.6 (0.3)	2.7 (0.3)	** < 0.001**	** < 0.001**	**0.01**
HAD-DEP (0–21)	3.3 (0.3)	4.1 (0.4)	1.4 (0.2)	1.4 (0.2)	** < 0.001**	0.63	0.19
PSS (0–40)	16.0 (0.6)	17.7 (0.9)	11.7 (0.6)	10.9 (0.8)	** < 0.001**	0.69	0.09
IBS-SSS (175–300)	228.8 (7.2)	217.2 (11.5)	NA	NA	NA	0.20	NA
Education							
Some HS	0	0	0	1 (1%)	0.06	0.45	0.36
HS graduate	1 (1%)	2 (4%)	6 (6%)	3 (4%)			
Some college	42 (33%)	15 (28%)	24 (26%)	13 (19%)			
College graduate	36 (28%)	13 (24%)	27 (29%)	22 (33%)			
Any post-graduate	49 (38%)	24 (44%)	37 (39%)	28 (42%)			
Diet							
American	72 (56%)	40 (74%)	48 (52%)	45 (67%)	0.26	**0.01**	**0.01**
Mediterranean	33 (26%)	4 (7%)	17 (18%)	10 (15%)			
Restrictive/vegetarian	19 (15%)	8 (15%)	24 (26%)	10 (15%)			
Missing	4 (3%)	2 (4%)	5 (5%)	2 (3%)			

Data are presented as mean (standard error) or number (%)

*HAD-ANX/HAD-DEP* Hospital Anxiety and Depression scale anxiety/depression, *HC* healthy control, *HS* high school, *IBS* irritable bowel syndrome, *IBS-SSS* IBS Severity Scoring System, *NA* not applicable, *PSS* Perceived Stress Scale

In total, 116/182 (64%) participants with IBS were classified as living in a neighborhood with high ADI, including 81/128 (63%) females with IBS and 35/54 (65%) males with IBS. In addition, 88/161(55%) HCs were classified as living in a neighborhood with high ADI, including 47/94 (50%) HC females and 41/67 (61%) HC males. Although high ADI was more frequent in all participants with IBS than in HCs, the difference did not reach significance (χ^2^ = 2.92, p = 0.09). Sex-specific comparisons revealed that high ADI was significantly more frequent in females with IBS than in female HCs (χ^2^ = 3.92, p = 0.048), but was similar in males with IBS and HC males (χ^2^ = 0.68, p = 0.68).

### ADI-brain morphology relationships

The analysis of ADI-brain morphology relationships revealed mainly negative associations between high ADI and surface area or cortical thickness in numerous brain regions, with the strength of the association dependent on sex, diagnosis, or both sex and diagnosis, as detailed below.

#### Disease-related differences

**Fig. 3:**
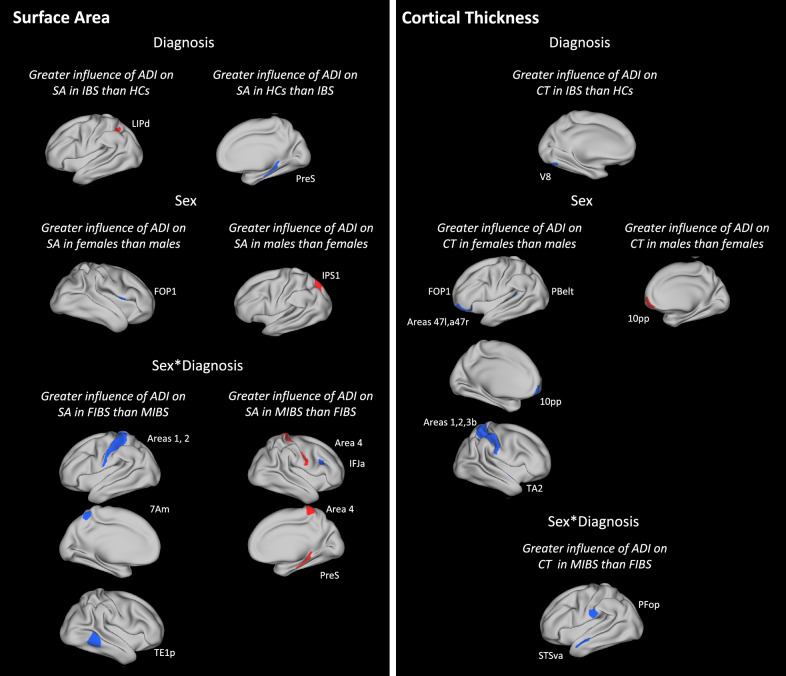
Relationships between high ADI and brain morphology parameters (surface area and cortical thickness). Relationships between ADI and brain alterations are presented according to (1) diagnosis (2) sex, and (3) sex*diagnosis. Red and Blue indicate positive and negative associations, respectively, that are greater in one group vs another group (as indicated in headings). Abbreviations for brain regions are provided in Tables 2 and 3. *ADI* area deprivation index. *CT* cortical thickness, *IBS* irritable bowel syndrome, *SA* surface area

Comparisons between participants in IBS and HCs revealed a significantly greater negative association between high ADI and surface area in right presubiculum (hippocampal subfield) in HCs than in participants with IBS and a significantly greater positive association between high ADI and surface area in left dorsal lateral intraparietal area in participants with IBS than in HCs (p’s < 0.01; Table 2; Fig. 3). Additionally, high ADI was negatively associated with cortical thickness in left visual area 8 to a significantly greater extent in participants with IBS than in HCs (p < 0.01; Table 3; Fig. 3).

**Table 2:** Brain regions showing associations between ADI and surface area according to diagnosis, sex, and sex*diagnosis

	HCPMMP Label	Region	Area description	BSR	P-value
HCs vs IBS					
More negative in HCs	R PreS	Presubiculum	Hippocampal subfield	2.61	0.009
IBS vs HCs					
More positive in IBS	L LIPd	Dorsal lateral intraparietal	Superior parietal	2.70	0.007
Females vs males					
More negative in females	R FOP1	Frontal operculum area 1	Frontal operculum	2.65	0.008
Males vs females					
More positive in males	L IPS1	Itraparietal sulcus area 1	Dorsal visual stream	2.84	0.005
FIBS vs MIBS					
More negative in FIBS	L 7Am	Anterior-medial 7	Superior parietal	2.71	0.007
More negative in FIBS	L 1	Area 1	Primary somatosensory	2.92	0.004
More negative in FIBS	L 2	Area 2	Primary somatosensory	2.59	0.01
More negative in FIBS	R TE1p	Posterior temporal area 1	Lateral temporal	2.92	0.004
MIBS vs FIBS					
More positive in MIBS	R 4	Area 4	Primary motor	2.70	0.007
More positive in MIBS	R PreS	Presubiculum	Hippocampal subfield	3.09	0.002
More negative in MIBS	R IFJa	Anterior inferior frontal junction	Dorsolateral prefrontal	2.78	0.005

*ADI* area deprivation index, *BSR* bootstrap ratio, *FIBS* females with IBS, *HC* healthy control, *IBS* irritable bowel syndrome, *L* left hemisphere, *MIBS* males with IBS, *R* right hemisphere

**Table 3:** Brain regions showing negative associations between ADI and cortical thickness according to diagnosis, sex, and sex*diagnosis

	HCPMMP label	Region	Area description	BSR	P-value
IBS vs HC					
More negative in IBS	L V8	Visual area 8	Ventral visual stream	2.78	0.005
Females vs males					
More negative in females	L 47 l	Lateral 47	Frontopolar	2.59	0.01
More negative in females	L a47r	Rostral anterior 47	Frontopolar	2.59	0.01
More negative in females	L 10 pp	10 polar-polar	Frontopolar	2.92	0.004
More negative in females	L FOP1	Frontal operculum area 1	Frontal operculum	2.70	0.007
More negative in females	L PBelt	Parabelt complex	Early auditory	2.74	0.006
More negative in females	R 3b	Area 3b	Primary somatosensory	2.78	0.005
More negative in females	R 1	Area 1	Primary somatosensory	3.12	0.002
More negative in females	R 2	Area 2	Primary somatosensory	2.59	0.01
More negative in females	R TA2	Temporal region A, area 2	Auditory association	2.63	0.009
Males vs females					
More positive in males	R 10 pp	Area 10 polar-polar	Frontopolar	2.63	0.009
MIBS vs FIBS					
More negative in MIBS	L PFop	Parietal operculum, area F	Inferior parietal	2.95	0.003
More negative in MIBS	L STSva	Ventral-anterior superior temporal sulcus	Auditory association	2.73	0.006

*ADI* area deprivation index, *BSR* bootstrap ratio, *FIBS* females with IBS, *HC* healthy control, *IBS* irritable bowel syndrome, *L* left hemisphere, *MIBS* males with IBS, *R* right hemisphere

#### Sex-related differences

Comparisons between male and female participants revealed a significantly greater negative association between high ADI and surface area in right frontal operculum area 1 in females than in males and a significantly greater positive association between high ADI and surface area in left intraparietal sulcus area 1 (p’s < 0.01; Table 2; Fig. 3). In addition, high ADI was negatively associated with cortical thickness in left anterior-rostral area 47, left frontal operculum area 1, right area 1, right area 2, and right area 3b to a significantly greater extent in females than in males and was positively association with cortical thickness in right area 10 polar-polar to a significantly greater extent in males than in females (p’s < 0.01; Table 3; Fig. 3).

#### Disease- and sex-related differences

The analysis of diagnosis*sex interactions revealed significantly greater sex differences in IBS than in HCs, with greater negative associations between high ADI and surface area in left anterior-medial area 7, right posterior temporal area 1, left area 1, left area 2, and right area 4 in females with IBS than in males with IBS; a greater positive association between high ADI and surface area in right presubiculum in males with IBS than in females with IBS; and a greater negative association between high ADI and surface area in right anterior inferior frontal junction in males with IBS than in females with IBS (p’s < 0.01; Table 2; Fig. 3). In addition, high ADI was negatively associated with cortical thickness in left parietal operculum area F and left ventral-anterior superior temporal sulcus to a significantly greater extent in males with IBS than in females with IBS (p’s < 0.01; Table 3; Fig. 3).

### ADI-symptom/microbial relationships

**Fig. 4:**
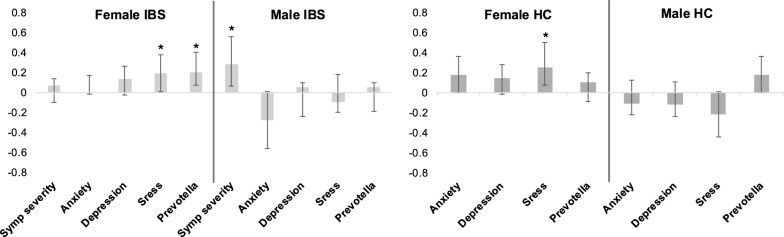
Relationships between high ADI and symptom/microbial parameters. High ADI is significantly associated with increased stress in HC and IBS females, symptom severity in IBS males, and *Prevotella* relative abundance in IBS females. *ADI* area deprivation index, *IBS* irritable bowel syndrome, *HC* healthy control

In the analysis of ADI-symptom/microbial relationships among participants with IBS, high ADI was significantly associated with greater IBS-SSS scores in males with IBS and greater PSS scores and *Prevotella* relative abundance in females with IBS (p’s < 0.01; Fig. 4). To better understand the lack of an association between high ADI and symptom severity in females with IBS, we performed a post hoc analysis of the relationship between IBS-SSS scores and PSS scores and IBS-SSS scores in females with IBS. The post hoc analysis revealed a trend toward a negative relationship between *Prevotella* relative abundance and IBS-SSS scores (general linear modeling with a gamma distribution and log-link function; coefficient = − 0.88, p = 0.07). In addition, the post hoc analysis revealed a significant positive association between PSS scores and IBS-SSS scores (partial correlation; r = 0.23, p = 0.04). In the analysis of ADI-symptom/microbial relationships among HCs, high ADI was significantly associated with greater PSS scores in female HCs, while no associations reached significance in male HCs (Fig. 4).

### Brain morphology-symptom/microbial relationships in IBS

**Fig. 5:**
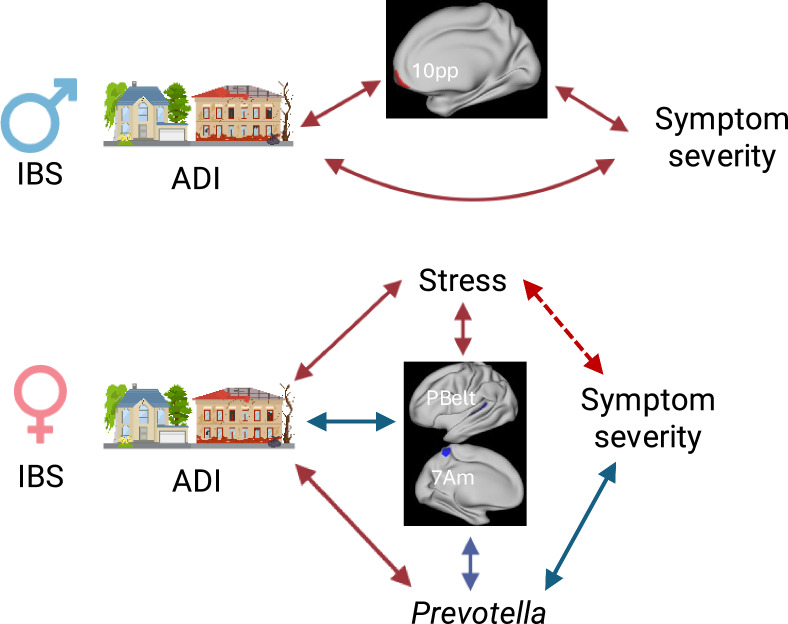
Summary of relationships between ADI-related morphological and clinical/microbial findings in IBS. High ADI is associated with increased cortical thickness in right area 10 pp in IBS males, which are also associated with increased symptom severity. In contrast, high ADI is significantly associated with decreased surface area in 7Am, which shows opposing relationships with stress and *Prevotella* relative abundance, in IBS females. High ADI is not associated with symptom severity in IBS females because high ADI is associated with increased stress and *Prevotella* relative abundance, which show opposing relationships with symptom severity. Significant associations are indicated with solid lines, trends are indicated with dashed lines. *ADI* area deprivation index, *IBS* irritable bowel syndrome, *PBelt* parabelt complex, *10 pp* area 10 polar-polar, *7Am* antero-medial area 7

We also evaluated relationships between cortical parameters identified in the ADI-brain morphology analysis and clinical/microbial parameters identified in the ADI-symptom/microbial analysis in males and females with IBS. For females with IBS, we included cortical parameters showing a greater influence of ADI in IBS vs HCs, females vs males, and females with IBS vs males with IBS. For males, we included cortical parameters showing a greater influence of ADI in IBS vs HCs, males vs females, and males with IBS vs females with IBS. In females with IBS, reduced surface area in left area 7Am (bootstrap ratio = 3.23, p = 0.001) and reduced thickness in the left parabelt complex (bootstrap ratio = 2.94, p = 0.002) were associated with greater *Prevotella* relative abundance and lower perceived stress. In males with IBS, increased cortical thickness of right area 10 pp (bootstrap ratio = 2.07, p = 0.04) was associated with greater IBS-SSS scores. A summary of all symptom/microbial relationships is provided in Fig. 5.

## Discussion

The present study evaluated associations between neighborhood disadvantage and clinical characteristics, cortical morphology, and *Prevotella* relative abundance in males and females with IBS and HCs. Consistent with our expectations, we found significant associations between high ADI and greater perceived stress and IBS symptom severity. However, these associations were sex-specific, with the former found only in females with IBS (as well as female HCs), and the latter in males with IBS. Additionally, high ADI was associated with greater *Prevotella* relative abundance in females, but not males, with IBS. We also found that high ADI was associated with alterations in surface area or cortical thickness in multiple regions, including regions involved in emotion regulation and pain processing, according to sex and/or diagnosis. Among these brain alterations, we found that reduced surface area of left area 7Am and thickness of the left parabelt complex were also associated with ADI-related clinical characteristics in females with IBS (i.e. *Prevotella* abundance and perceived stress), while increased thickness of right area 10 polar-polar (10 pp) was associated with ADI-related clinical characteristics in males with IBS (i.e. symptom severity).

Overall, the results suggest that high ADI is associated with more severe symptoms and greater morphological alterations in IBS-implicated regions involved in cognitive control and pain processing in males with IBS. In contrast, the results also suggest that ADI has multi-faceted effects in females with IBS, including higher levels of risk factors that may exacerbate IBS symptoms such as perceived stress, as well as protective factors, such as *Prevotella* relative abundance, which has been shown to be negatively associated with symptom severity [26].

### ADI-cortical morphology relationships

The evaluated morphological parameters in the present study comprised cortical thickness and surface area. These two parameters have distinct genetic and behavioral correlates and should be considered separate morphometric features, unlike cortical volume, which reflects a combination of thickness and surface area [39, 40]. Reductions in cortical surface area may reduce computational capacity and functional specificity [45]. Reductions in cortical thickness may reflect cortical pruning, improving efficiency; however, it may also reflect excessive apoptosis, disrupting function [45]. Chronic stress, including psychosocial stress, is associated with accelerated brain aging, resulting in decreases in surface area and cortical thickness [46, 47]. Thus, the observed reductions in surface area and cortical thickness with high ADI may reflect stress-related brain aging.

Few regions showed differences in high ADI-morphology associations according to diagnosis regardless of sex. Dorsal lateral intraparietal area was one of these regions, with a significantly greater positive association between surface area and high ADI in participants with IBS than in HCs. The lateral intraparietal area is involved in spatial saliency, guiding eye movements and visual attention, and modulating sensory-motor interactions [48]. This area has been implicated in anxiety and unnecessary attention toward threat [49, 50]. Thus, the association between high ADI and the dorsal lateral intraparietal morphology IBS may relate to an attentional bias toward threat in IBS [51]. In addition, the presubiculum showed a significantly greater negative association between morphology and high ADI in HCs than in participants with IBS. However, this region also showed a significant sex*diagnosis interaction, with a greater positive association with ADI in males with IBS than in females with IBS. The presubiculum is a hippocampal subfield involved in spatial navigation [52]. Data from patients with Cushing’s Disease (a neuroendocrine disorder) suggest that the presubiculum volume is particularly sensitive to chronic cortisol overexposure [53], and neighborhood disadvantage is associated with higher cortisol levels [54, 55]. Thus, the finding of a greater negative association between ADI and presubiculum volume in HCs vs IBS and positive association in males with IBS vs females with IBS may be related to differences in the impact of ADI on hypothalamic pituitary adrenal axis function. However, additional research is needed.

A greater number of regions showed differences in ADI-morphology associations according to sex (especially cortical thickness) or sex*diagnosis (especially surface area), suggesting the importance of considering sex in evaluating relationships between neighborhood disadvantage and brain structure in healthy and pain populations. The morphology (especially cortical thickness) of regions involved in emotion regulation and pain processing, including bilateral frontopolar (involved in integrating emotional context and goal-directed actions) [56], bilateral frontal operculum cortices (involved in the perception and expression of emotion) [57, 58], and right primary somatosensory (involved in pain perception and modulation) [59], was more negatively associated with ADI in females than in males, regardless of diagnosis. Thus, ADI may generally have a greater impact on emotion and pain processing in females than in males, which we speculate may reflect greater vulnerability to the development of IBS, and other pain conditions, in females living in disadvantaged neighborhoods. However, larger-scale studies are needed to elucidate relationships between neighborhood disadvantage and the risk of developing IBS.

We also found that some sex differences were more pronounced in IBS than in HCs. Females with IBS showed greater negative associations between ADI and surface area in left primary somatosensory cortices than males with IBS. In contrast, males with IBS showed greater negative associations between ADI and thickness/surface area in dorsolateral prefrontal (involved in involved in cognitive-emotional control) [60, 61], parietal operculum (involved in pain modulation) [62], and superior temporal cortices (involved in socioemotional awareness) [63]. These results are consistent with previous findings of more prominent somatosensory alterations in females with IBS, whereas males with IBS may show more cognitive control alterations [5, 64, 65].

In the present dataset, the frequency of major dietary categories (American, Mediterranean, vegetarian/restrictive) did not significantly differ between high and low ADI groups (overall and within sex and diagnosis subgroups; p-values ranging 0.17–0.85). Further, controlling for diet had minimal impact on the morphology results (affecting only the R frontal operculum area 1 and left area 2 findings in Table 2). However, a more-fine-grained analysis of the role of dietary intake (e.g. diet quality, fat content) on relationships between ADI and brain morphology should be considered in future studies.

### ADI-clinical/microbial relationships

We found that high ADI was associated with higher IBS-SSS scores in males with IBS, suggesting that neighborhood disadvantage exacerbates symptoms in males with IBS. In contrast, ADI was not associated with IBS-SSS scores in females with IBS. Rather, high ADI was associated with greater *Prevotella* relative abundance and perceived stress in females with IBS. Gut microbiota composition is influenced by individual lifestyle factors such as diet and exercise; however, these factors are also dependent on neighborhood conditions such as the amount of greenspace and type of foods readily available [22]. Gut microbiome profiles associated with neighborhood disadvantage could contribute to health disparities, including disparities in chronic disease outcomes, such as IBS. Greater neighborhood disadvantage is associated with lower microbiota diversity [22, 66]. In addition, several studies have reported an association between neighborhood disadvantage with increased *Prevotella* abundance [21, 22]. Thus, the present results are consistent with a positive influence of ADI on *Prevotella* abundance, but suggest that the relationship is more robust in females with IBS. In our previous work in a larger sample of participants with IBS with microbiota data (n = 336 vs n = 113), we found that lower *Prevotella* relative abundance was significantly associated with higher IBS symptom severity in females, but not males, with IBS [26]. Consistent with this, there was a trend toward a negative relationship between *Prevotella* relative abundance and IBS-SSS scores in the present sample of females with IBS. Additionally, there was a significant positive association between PSS scores and IBS-SSS scores. Thus, the lack of an association between ADI and IBS symptom severity in females with IBS may be related to opposing influences of ADI-related increases in perceived stress and *Prevotella* relative abundance on symptom severity in females with IBS (i.e. *Prevotella* may buffer the effect of ADI-related stress on symptom severity in females with IBS), as depicted in the lower left portion of Fig. 1. These results suggest that sex-specific protective factors (i.e. *Prevotella* relative abundance) may exist in disadvantage neighborhoods that could be exploited to improve the clinical course. Further, factors driving symptom severity may differ by neighborhood advantage in females with IBS, with increased stress as an important contributing factor in neighborhoods with high disadvantage and lower *Prevotella* abundance in neighborhoods with low disadvantage.

We also found that high ADI was associated with higher PSS scores in female HCs, but not in male HCs, similar to the findings in participants with IBS. As the PSS is a measure of stress in the prior month, these results suggest that ADI has less effect on the perception of ongoing stress in males compared to females even though both sexes may show consequences of ADI as a chronic stressor.

### Connecting ADI-related morphological and clinical/microbial findings in IBS

High ADI had a greater negative association with the surface area of area 7Am in females than in males with IBS and a greater negative association with thickness of the left parabelt complex in females than in males (see Tables 2 and 3). In females with IBS, greater reductions in these two cortical areas were also associated with both lower perceived stress and greater *Prevotella* relative abundance. Thus, these regions may play a role in the protective attributes of *Prevotella* in females with IBS (potentially buffering ADI-related stress effects as mentioned above). Moreover, larger surface area/cortical thickness in these areas (characteristic of females with IBS with low ADI) are associated with a worse clinical/microbial profile. Area 7Am is a subregion of the precuneus in parietal cortex (specifically, it is anterior dorsal precuneus), with widespread connections to multiple resting-state networks, including the default mode, sensorimotor, and dorsal attention networks, as well as emotion regulation regions [67]. It is involved in multisensory integration and bodily awareness [68], and plays a role in stress perception [69, 70]. The parabelt complex is tertiary auditory cortex that also shows connectivity with regions in default mode and sensorimotor networks [71]. In a recent neuroimaging study examining brain regions coupled to the gastric rhythm during rest, left area 7Am and parabelt complex were both part of the identified gastric network, suggesting their importance in interoceptive processing of gastric activity [72], which shows alterations postprandially in some patients with IBS [73]. Further, variation in parietal cortex surface area is associated with variation in perceptual thresholds (larger surface area, lower duration threshold) [74]. We speculate that morphological variation in this region also influences perceptual processing of gastrointestinal stimuli, as well as stressful experiences.

High ADI had a greater positive association with cortical thickness in right area 10 pp in males than females (see Table 3). In males with IBS, greater increases in right area 10 pp was also associated with greater symptom severity. Area 10 pp is a subregion of the frontopolar cortex and is involved in higher-order cognitive functions [75]. Decreased testosterone level due to androgen deprivation therapy for prostate cancer is associated with increased frontopolar cortical thickness, which mediates declines in working memory performance [76]. Patients with IBS show reduced testosterone levels, associated with increased symptom severity [77]. Thus, we speculate that high ADI may be differentially associated with testosterone levels in males with IBS, affecting area 10 pp morphology and function; however, further research incorporating sex hormone data is needed.

### Limitations

In the present study, we focused on exclusively on *Prevotella* relative abundance based on our previous work [26]; however, other microbial factors may be shaped by ADI, with associated changes in brain structure/function and clinical profiles. Although we controlled for diet, we did not control for other lifestyle factors, such as physical activity and sleep quality, that could influence both the gut microbiome and brain morphology. Moreover, in addition to increased stress, neighborhoods disadvantage is associated with increased rates of infection and food insecurity, which may contribute to a higher rate of post-infection IBS, which could have influenced the results; however, we did not have reliable data on the incidence of post-infection IBS in the present dataset. Additionally, the subgroup analyses may have been statistically underpowered, especially for comparisons involving males with IBS, due to relatively small sample sizes. Further, we included both Rome III- and Rome IV-positive participants, introducing heterogeneity. Additionally, although neighborhoods in Southern California range in ADI, the ADI is generally lower compared to that in other areas of the nation, as reflected in the skewed ADI distribution in the present study. Therefore, the results may not generalize to individuals with more severe ADI and should be interpreted with caution. Finaly, as a major limitation of cross-sectional studies, causality could not be addressed.

A larger-scale study, with more diverse neighborhoods and longitudinal data, is needed to better understand role of ADI in the development and clinical course of IBS. However, the present study provides new information regarding the potential of ADI to differentially impact the brain–gut–microbiome axis in males and females with IBS. We hope that the present study will inspire future research to better understand the relationships between neighborhood context, microbial composition and function, and brain structure and function in males and females with IBS. Since IBS is a disorder of gut–brain interactions and is influenced by stress, this line of investigation is consistent with the conceptual model and literature on IBS.

## Conclusions

Neighborhood disadvantage, a social determinant of health, may be considered as a chronic stressor that influences brain morphology and clinical and microbial characteristics in IBS. Specifically, neighborhood disadvantage is associated with greater symptom severity in males with IBS, while in females with IBS, it is associated with higher perceived stress and *Prevotella* abundance in females with IBS, which have opposing influences on symptom severity, suggesting that both risk (stress) and protective factors (*Prevotella*) may exist in neighborhoods with high disadvantage. Further, neighborhood disadvantage generally has a greater negative impact on emotion/pain-related cortical morphology in females than in males, which may affect lifetime risk for IBS. Neighborhood disadvantage is also associated with greater sex differences in IBS, with more prominent somatosensory alterations (involved in processing pain) in females with IBS and prefrontal/parietal alterations (involved in cognitive control, pain modulation) in males with IBS. ADI-related alterations in the morphology of left anterodorsal precuneus may be related to protective characteristics of *Prevotella* under high disadvantage in females with IBS, while alterations in frontopolar cortex may contribute to symptom severity in males with IBS. These findings highlight the interplay between social and biological factors in IBS and underscore the need for targeted, sex-specific interventions.

## Data Availability

Because the data presented is part of several ongoing projects, availability of data will be made available by request.
